# Classifying Patient Characteristics and Determining a Predictor in Acute Stroke Patients: Application of Latent Class Analysis in Rehabilitation Practice

**DOI:** 10.3390/jcm14155466

**Published:** 2025-08-04

**Authors:** Junya Uchida, Moeka Yamada, Hirofumi Nagayama, Kounosuke Tomori, Kohei Ikeda, Keita Yamauchi

**Affiliations:** 1 Dalla Lana School of Public Health, University of Toronto, Toronto, ON M5S 1A1, Canada; 2 Department of Occupational Therapy, Shinyokohama Rehabilitation Hospital, Yokohama 221-0864, Japan; 3 Department of Occupational Therapy, Kanagawa University of Human Services, Yokosuka 238-8522, Japan; 4 Major of Occupational Therapy, Department of Rehabilitation, School of Health Sciences, Tokyo University of Technology, Tokyo 144-0051, Japan; 5 Graduate School of Health Management, Keio University, Fujisawa 252-0883, Japan; 6 Faculty of Nursing and Medical Care, Keio University, Fujisawa 252-0883, Japan

**Keywords:** rehabilitation, acute stroke, latent class analysis, patient characteristics, physical therapy, occupational therapy, speech therapy

## Abstract

**Background/Objectives**: Predicting comprehensive patient characteristics is essential for optimal individualized rehabilitation plans for acute stroke patients. However, current models primarily predict single outcomes. This study aimed to assess the applicability of latent class analysis (LCA) in rehabilitation practice by identifying comprehensive characteristics and associated predictors in acute stroke patients. **Methods**: We conducted a retrospective observational study using the Japan Association of Rehabilitation Database, including 10,270 stroke patients admitted to 37 acute-care hospitals between January 2005 and March 2016. Patients were classified using LCA based on outcomes at discharge, including Functional Independence Measure (FIM), National Institutes of Health Stroke Scale (NIHSS) subscales for upper-extremity function, length of hospitalization, and discharge destination. Predictor variables at admission included age, FIM scores, NIHSS subscales for upper-extremity function, stroke type, and daily rehabilitation volume. **Results**: 6881 patients were classified into nine distinct classes (class size: 4–29%). Class 1, representing the mildest cases, was noted for independent ambulation and good upper limb function. Class 2 comprised those with the most severe clinical outcome. Other classes exhibited a gradient of severity, commonly encountered in clinical practice. For instance, Class 7 included right-sided paralysis with preserved motor activities of daily living (ADLs) and modified dependence in cognitive functions, such as communication. All predictors at admission were significantly associated with class membership at discharge (*p* < 0.001). **Conclusions**: LCA effectively identified unique clinical subgroups among acute stroke patients and demonstrated that key admission variables could predict class membership. This approach offers a promising insight into targeted, personalized rehabilitation practice for acute stroke patients.

## 1. Introduction

Annually, 15 million people worldwide suffer a stroke, leading to five million deaths and another five million individuals left with stroke-related disabilities [1]. Rehabilitation services are vital as they foster functional recovery and independence in patients with acute stroke [2]. A formal assessment of activities of daily living (ADLs), instrumental ADLs, communication abilities, and functional mobility is recommended for the rehabilitation of stroke patients before discharge, and the findings should be incorporated into the care transition and discharge planning [2,3]. In addition, clinicians consider a patient’s prognosis of functional recovery as the most critical factor when considering a discharge destination from the acute setting [4,5]. Therefore, predicting a patient’s condition at discharge based on assessment results at admission can provide a valuable contribution to personalized rehabilitation plans.

A recent review [6] reported that several tools have been developed to predict outcomes for independence [7,8,9], upper-extremity function [10,11], gait [12,13,14,15,16,17], and swallowing [18,19]. These previous studies predicted a single outcome based on multiple variables. However, from a clinical perspective, it is crucial to predict comprehensive patient characteristics by incorporating multiple outcomes at discharge, not just a single outcome. Comprehensive patient characteristics might be, for example, that a patient is expected to be able to walk independently but may need assistance with communication and cognitive function. While the clinical relevance of predicting patient characteristics based on multiple outcomes is well recognized, few studies have addressed how to classify such profiles at discharge or predict them from admission data. Filling this gap is essential for individualized rehabilitation planning.

Latent class analysis (LCA) has the potential to solve these problems. LCA is a subset of structural equation modeling that categorizes heterogeneous classes (segments) of the study population by associating manifest variables with notions (latent variables) that cannot be directly measured [20]. Previous studies have incorporated LCA to understand the potential characteristics of a population with the capability to predict latent class membership [21]. For instance, LCA is a feasible method to build an atrial fibrillation risk model, despite the heterogeneity in risk factors among individuals at risk [22].

However, there are few studies on the application of LCA to rehabilitation practice. Using LCA, it is possible to identify classes of comprehensive patient characteristics (such as discharge destination, physical function, functional abilities, and length of stay) and to determine the predictors for which class a patient belongs based on their characteristics at admission. Therefore, this consideration will provide a new solution to a problem that previous prediction models have not solved.

The purpose of this study was to examine the applicability of LCA to rehabilitation practice by identifying the LCA model classifications for discharged acute stroke patients and determining the variables at admission that were predictive of LCA model classifications.

## 2. Materials and Methods

### 2.1. Study Design

We adopted a retrospective observational study approach, consisting of two main analyses aligned with the study’s objectives. The first involved statistically classifying patient characteristics at discharge via LCA, while the second focused on investigating variables at admission to predict membership in these classified classes. The study protocol was developed and conducted following the STROBE (Strengthening the Reporting of Observational Studies in Epidemiology) guidelines [23].

### 2.2. Database

A robust sample size and a comprehensive database representing a diverse range of patient characteristics were essential for this study. Consequently, we utilized the Japan Association of Rehabilitation Database (JARD) [24,25], which collected data voluntarily from patients admitted to participating hospitals between January 2005 and March 2016. This database has been utilized in several previous studies [24,25,26]. It includes records of the National Institutes of Health Stroke Scale (NIHSS) and Functional Independence Measure (FIM) scores, alongside patient demographics such as age, stroke type, rehabilitation type, length of hospitalization, and additional relevant variables. In 2016, data were collected from 33,657 patients across 80 participating hospitals. Our analysis focused on data gathered from stroke patients admitted to acute-care hospitals (37 hospitals, *n* = 10,270) between admission and discharge. For the purposes of this study, acute patients were defined as those admitted within 7 days after stroke onset, and acute-care hospitals were defined as the facilities where patients were initially admitted after stroke.

In October 2021, JARD approved access to data for this study. Individual participants were identified and analyzed between June and December 2022.

### 2.3. Participants

The following patients were included in this study: (1) patients with acute stroke admitted to registered hospitals in the Japan Association of Rehabilitation Database between 2005 and 2015, (2) those aged ≥18 years, and (3) those provided with rehabilitation (physical, occupational, and speech therapy).

The exclusion criteria were (1) no data on age at admission; (2) no data on discharge destination; (3) age <18 years; (4) no rehabilitation provided during hospitalization; (5) duration from stroke onset to admission of >7 days; (6) rehabilitation for >9 units (180 min) daily during hospitalization because, in the Japanese rehabilitation system, there is a limit of 9 units daily for rehabilitation; therefore, assuming that a patient receiving >9 units daily is undergoing a normal rehabilitation program is difficult; (7) length of stay of <1 day or >179 days; (8) no data on all items of the FIM and NIHSS at admission and/or discharge; and (9) no data on all items of the FIM both at admission and discharge.

### 2.4. Statistical Analyses

We used Latent GOLD 6.0 (Statistical Innovations, Arlington, MA, USA) for LCA and predicting which class a patient belongs to based on their characteristics (variables) at admission with a multinomial logistic regression model. We conducted the two-step method proposed by Bakk and Kuha with default settings in Latent GOLD 6.0 [27]. When classifying patient characteristics and identifying predictors, this method has less bias than other methods and is recommended even when missing values exist [28,29,30]. The two-step method comprises the following steps: (1) fitting the latent class measurement model on its own and (2) maximizing the joint likelihood with the measurement model’s parameters and exogenous latent variables’ parameters fixed at the first step’s estimated values, allowing only the structural model’s remaining parameters to be estimated in the second step [27]. The optimal latent model for the number of latent classes was determined using a combination of Bayesian information criterion (BIC), entropy, Vuong–Lo–Mendell–Rubin, and class size [31,32]. We processed missing data by employing full information maximum likelihood default in Latent GOLD 6.0 [33,34]. The significance level was set at *p* < 0.05.

### 2.5. Outcome Variables

We conducted an LCA with our selected outcome variables to identify patient characteristics at discharge. Prior to conducting any data analysis, we a priori selected functional abilities, cognitive functions, upper-extremity function, length of hospitalization, and discharge destination as outcome variables for classification. These variables were chosen based on a comprehensive review of previous studies and our clinical perspectives to ensure clinical relevance and comparability with existing literature. LCA is the label given to a form of finite mixture modeling in which all observed indicators are categorical [35]; therefore, continuous variables were transformed into categorical variables.

Functional abilities at discharge were referred to using the FIM. The FIM includes 18 items to determine the amount of assistance needed by a person with a disability to perform daily activities safely and effectively [36]. All items were scored using a 7-point scale: 1, total assistance; 2, maximum assistance; 3, moderate assistance; 4, minimal assistance; 5, supervision; 6, modified independence; and 7, complete independence. Based on previous studies [37,38], we classified these seven scales into three groups: complete dependence (1–2), modified dependence (3–5), and independence (6–7). Outcome variables extracted as functional abilities were eating, transfers (bed/chair/wheelchair), toileting, locomotion (walking/wheelchair), and transfers (shower/bathtub).

Cognitive functions were also described, referring to the cognitive subscale of the FIM. Three items of the FIM cognitive subscale were extracted: comprehension, expression, and social interaction. Social interaction was identified as a significant predictor of post-rehabilitation destinations in patients with acute stroke [39]. Patients with comprehension deficits could have serious problems in their homes despite family support because they may not be able to understand the provided information [40].

Information on upper-extremity function was collected based on the score on the motor arm, one of the NIHSS subscales. The NIHSS rates deficits and is widely used in modern neurology [41]. The score of the motor arm ranges from 0 to 4. A high score on this item indicates severe impairment in a patient’s upper extremities.

The length of hospitalization varies between patients depending on stroke severity, the patient’s background influencing their discharge destination, and other factors. Therefore, it was classified into four categories: 1–14 days, 15–28 days, 29–42 days, and ≥43 days.

### 2.6. Predictor Variables

The predictor variables were selected a priori, based on prior literature and clinical reasoning. These included patient demographics and functional abilities at admission, which were expected to predict class membership at discharge according to existing evidence, relevant literature, and our clinical perspective. The variables included age, functional abilities, comprehension, upper-extremity function, type of stroke, and daily amount of rehabilitation (physical, occupational, and speech therapies) during hospitalization.

We included the following functional outcomes in FIM items: eating [39,42], locomotion (walk/wheelchair) [43], toileting [42], and comprehension [40,44]. Stroke patients had a higher possibility of being discharged home if admitted with a higher motor FIM score and a lower chance if they were older or had a cognitive defect [45]. FIM toileting was one of the initial items that predicted disposition in acute stroke patients [42]. Based on the motor FIM score at admission, there was a correlation between locomotion gain and other self-care in stroke patients. A variation in locomotion gain influenced the improvement in self-care [43]. Aphasia was also identified as a strong prognostic factor in post-stroke outcomes [44]. Furthermore, deficits in auditory comprehension, reading comprehension, and oral spelling to dictation were associated with a higher possibility of discharge to a setting other than home [40].

The use of the NIHSS score as an early predictor of outcome following acute hospitalization for stroke has received support [46]. The NIHSS score of the affected upper extremity assessed at admission was associated with long-term functional outcomes evaluated using the FIM in patients with middle cerebral artery infarction [47].

The stroke types confirmed in the dataset were grouped into the following categories: lacunar infarction, atherothrombotic cerebral infarction, cardiogenic embolism, cerebral infarction (others/unknown), hypertensive cerebral hemorrhage, cerebral hemorrhage (others/unknown), subarachnoid hemorrhage, and others/unknown.

Regarding the amount of daily rehabilitation, according to Japanese health insurance guidelines, 20-minute rehabilitation (physical, occupational, and speech therapies) is defined as 1 unit. This study categorized the amount of daily rehabilitation provided for each patient available on the dataset into four groups: <2 units (<40 min), ≥2–<4 units (40–80 min), ≥4–<6 units (80–120 min), and ≥6 units (>120 min).

## 3. Results

### 3.1. Patient Characteristics

**Figure 1:**
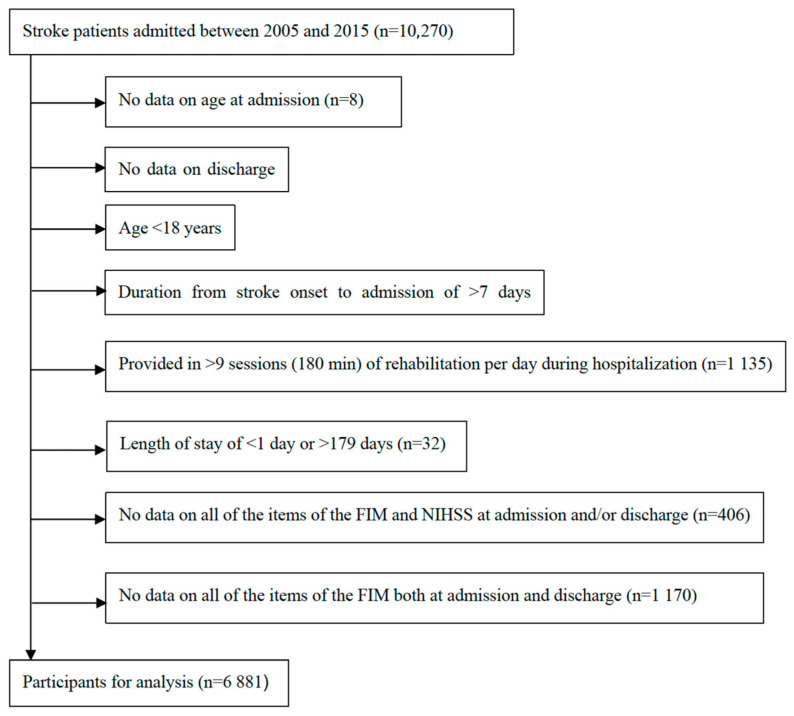
Flow diagram of the patient selection process.

After applying the inclusion and exclusion criteria in this study, 6881 patients were included (Figure 1), and baseline characteristics at admission and discharge are summarized in Table 1. The mean age at admission was 73.7 years, and the average length of stay was 29.5 days. The total FIM scores, scores of all FIM subitems, and scores of the NIHSS improved at discharge from admission.

### 3.2. Latent Classes of Patient Characteristics at Discharge

A total of 6881 patients were classified using LCA based on their outcomes. Latent class models ranging from 1 to 12 classes were examined to select the optimal one, with model-fit statistics displayed in Table 2. The decline in BIC/AIC fit between the models began with the nine-class model, which also had the smallest class size of less than 5%. Considering the interpretability of the class characteristics and the recommendation for a minimum class size of at least 5%, we opted for the nine-class model for this study [32].

Table 3 shows the class sizes and item response probabilities for the outcomes at discharge within each class. For example, a class size of 29% for Class 1 indicates that 29% of all patients belong to this class and that 97% of the patients within Class 1 are predicted to return home as “discharge destination.” The overall characteristics of each class indicated that Class 1 was the mildest (with independence in ADL and higher upper-extremity function, with a shorter length of stay and the highest possibility of home discharge), while Class 2 was the most severe (complete dependence in ADL and lower upper-extremity function, with a longer length of stay and the highest possibility of non-home discharge, including discharge to another hospital, discharge to a care facility, or in-hospital death). Different gradations characterized classes 3–9; these patient characteristics were clinically acceptable.

### 3.3. Predictors of Class Membership

Table S1 presents the odds ratios (ORs) and 95% confidence intervals (CIs) from a multinomial logistic regression model predicting latent class membership with Class 1 being the reference. All predictors of patient characteristics at admission were statistically significant enough to predict outcomes at discharge (*p* < 0.01). Relatively stronger predictors relative to Class 1 (maximum odds ratio was >10.0 in a single predictor) were the right/left motor arms of the NIHSS score. Class 8 showed extremely high odds of belonging if the left motor arm of the NIHSS score was 4. Class 9 also showed particularly high odds if the right motor arm of the NIHSS score was 4. Furthermore, regarding the amount of daily rehabilitation, the odds ratio tended to decrease in the severe class and increase in the mild class as the amount of rehabilitation increased. However, this tendency was not necessarily observed in several classes. Model fit indices and classification diagnostics for the latent class model are presented in Table S2.

### 3.4. Model Application

As an application of this study’s findings, we demonstrate a model predicting comprehensive patient characteristics at discharge based on variables at admission (created using Excel for Mac, Version 16; see Video S1). The predicted results can allow practitioners to provide the appropriate intensity of rehabilitation that maximizes effectiveness. Our made-up example for the model application is a patient aged 85 years with a cardiogenic embolism and a severe disability on the right side of the body. The patient is provided 4–6 units (80–120 min) of rehabilitation daily and requires modified to complete assistance with ADLs. According to the predicted probability, the patient has the highest possibility of non-home discharge and right moderate upper extremity disability at discharge. The patient is predicted to require complete assistance with ADLs and complete assistance with communication and social interaction.

## 4. Discussion

In this study, we applied LCA to classify discharged acute stroke patients and to determine which admission variables predicted these classifications. The analysis revealed that patient characteristics at discharge could be grouped into nine distinct classes. Additionally, all selected predictors at admission were significantly associated with these class memberships. These results suggest that LCA could be effectively applied in rehabilitation practice, providing insights that single-outcome predictions typically do not capture. We highlight two key aspects to illustrate this point.

The latent class analysis (LCA) in this study revealed clinically meaningful discharge profiles that aligned closely with practitioners’ expectations. Notably, differences were observed between the classes that predominantly included patients with left upper extremity paralysis (Classes 6 and 8) and those with right-sided paralysis (Class 9). While such lateralized functional differences have been widely reported in the literature [48,49,50], our findings demonstrate how these neurological patterns emerge within broader, multidimensional patient profiles that incorporate ADL function, communication ability, discharge destination, and length of hospital stay. For example, patients in Classes 6 and 8 tended to show more assistance needs in ADLs and longer hospital stays while maintaining relatively higher communication ability. This profile may reflect the influence of spatial neglect associated with right hemisphere damage, which affects independence in daily activities but not language function. In contrast, Class 9 was characterized by lower communication function despite relatively preserved ADL performance and showed a greater likelihood of non-home discharge. These patterns may relate to aphasia caused by left hemisphere damage, which can complicate communication and social reintegration even when motor function is relatively preserved. These distinctions, derived from multiple co-occurring indicators, support the value of LCA in identifying clinically relevant subgroups that extend beyond single-variable classifications.

It is important to acknowledge that each class included a range of patient characteristics, and no group was entirely homogeneous. However, the observed probabilistic patterns across classes offered a structured and intuitive understanding of patient heterogeneity. This approach provides a useful framework for anticipating care needs and developing individualized rehabilitation plans early in the hospitalization period.

The second point relates to the amount of daily rehabilitation. In this study, we included the amount of daily rehabilitation as one of the predictor variables, allowing us to estimate how varying levels of intensity may influence a patient’s probability of belonging to specific discharge classes. These classes incorporate multiple outcomes such as upper extremity function, ADL performance, communication ability, discharge destination, and length of hospital stay.

While our model does not determine the causal effects of rehabilitation intensity, it provides a framework for simulating how patient outcomes may vary depending on both their admission characteristics and the amount of therapy provided. This approach may offer useful insights into tailoring rehabilitation strategies in the acute phase of stroke care.

Previous studies have questioned the widely accepted notion that “more practice is always better,” particularly in the early days after stroke onset [51]. Our findings also suggest that higher rehabilitation intensity does not necessarily correspond to milder discharge classes. By incorporating rehabilitation intensity into the prediction model, our study demonstrates the potential to estimate an appropriate amount of daily rehabilitation tailored to individual patient characteristics.

The strengths of this study include the application of LCA to classify the comprehensive patient characteristics that incorporate multiple outcomes at discharge. This classification has scientifically clarified the comprehensive patient characteristics at discharge. Furthermore, the model enables simulation of how patients with different admission characteristics may respond to varying levels of rehabilitation intensity. This capability may support clinicians in developing tailored rehabilitation strategies from the early phase of stroke care. Beyond clinical applications, such predictive models may also inform health policy by supporting a transition from standardized rehabilitation provision toward personalized, needs-based rehabilitation delivery. These simulations are made possible by the inclusion of admission-based predictors, highlighting the potential of early assessment data in shaping individualized care plans.

Our study has some limitations. First, the dataset we referred to did not contain sufficient details about interventions provided for participants. Hence, the intervention differences among participants might have influenced the LCA and prediction model results. Second, other potential predictors that could more significantly influence outcomes at discharge might be available at admission. However, we selected predictor variables according to the findings of as many previous studies as possible and our clinical viewpoint, considering usefulness and applicability. Therefore, we consider the model the most applicable presently. The model will be improved if these issues are addressed in future validation studies. Third, the absence of psychosocial variables such as psychological distress, social support, and patient engagement, which are known to affect rehabilitation outcomes, is another important limitation. These important factors were not available in the current dataset, but future studies should consider their inclusion to enhance the model’s comprehensiveness and clinical relevance. In addition, the current model does not incorporate environmental or social determinants, such as socioeconomic status, cultural background, and geographic location, which may influence rehabilitation outcomes and class membership. Future research should aim to integrate these contextual variables and adopt an intersectional perspective to enhance the model’s explanatory power and equity focus. Such an approach aligns with the Learning Rehabilitation System framework, which emphasizes continuous learning and adaptation across levels of rehabilitation service provision [52]. Furthermore, future development of the model could benefit from alignment with the World Health Organization’s International Classification of Functioning, Disability and Health (ICF) framework. This would allow for a more holistic assessment by addressing not only physical and cognitive functioning but also environmental factors, personal attributes, and participation in community life [53]. Lastly, while this study has demonstrated that Latent Class Analysis (LCA) can be effectively applied for predicting comprehensive outcomes in stroke rehabilitation patients, it is not our intention to assert that the division into nine classes is definitive. Naturally, the results are dependent on the dataset used, and thus, the applicability of our findings should be primarily seen as evidence of LCA’s potential usefulness in the rehabilitation field.

In conclusion, we conducted a classification of characteristics of patients with acute stroke, incorporating multiple outcomes at discharge, and investigated the predictors for class membership to determine whether LCA can be applied to rehabilitation practice. Comprehensive patient characteristics at discharge were classified into nine classes, and all chosen predictors at admission had significant associations with class memberships. These results suggest that LCA could be applied in rehabilitation practice and is expected to be widely used. Further studies could shed light on the potential influence of the differences in interventions on outcomes and the appropriate amount of rehabilitation for stroke patients depending on their medical characteristics and severity.

## Figures and Tables

**Table 1:** Patient characteristics of this study.

	Overall *n* = 6801	
**Category variable**	***n* (%)**	
**Sex,** Female	3046 (44.3%)	
**Type of stroke**		
Lacunar infarction	1091 (15.9%)	
Atherothrombotic cerebral infarction	1697 (24.7%)	
Cardiogenic embolism	1138 (16.5%)	
Cerebral infarction (others/unknown)	614 (8.9%)	
Hypertensive cerebral hemorrhage	1169 (17.0%)	
Cerebral hemorrhage (others/unknown)	400 (5.8%)	
Subarachnoid hemorrhage	339 (4.9%)	
Others/Unknown	60 (0.9%)	
Missing	373 (5.4%)	
**Amount of rehabilitation per day**		
Less than 2 units (less than 40 min)	2277 (33.1%)	
2 or more to less than 4 units (40–80 min)	2329 (33.8%)	
4 or more to less than 6 units (80–120 min)	990 (14.4%)	
6 or more units (more than 120 min)	1285 (18.7%)	
**Continuous variable**	**Mean (SD)**	
**Age**	73.7 (12.7)	
**Length of stay, days**	29.5 (21.4)	
	**Admission**	**Discharge**
**Motor-FIM** (admission: *n* = 6751, discharge: *n* = 6652)	33.4 (23.5)	57.0 (29.7)
**Cognitive-FIM** (admission: *n* = 6774, discharge: *n* = 6676)	20.8 (11.4)	24.1 (10.7)
**FIM (total)** (admission: *n* = 6821, discharge: *n* = 6726)	54.2 (32.6)	80.9 (39.1)
**NIHSS (total)** (admission: *n* = 6575, discharge: *n* = 6544)	8.3 (8.9)	6.2 (8.2)

FIM, Functional Independence Measure (range, 18–126, higher = better); NIHSS, National Institutes of Health Stroke Scale (range, 0–42, higher = worse); *n*, number of participants; SD, standard deviation.

**Table 2:** Model-fit statistics for latent class analysis models with 1 to 12 classes.

	LL	BIC	ΔBIC	AIC	ΔAIC	df	VLMR	Entropy R^2^	Smallest Class Size
1-Class	−82,978	166,230	-	166,018	-	6858		1.00	-
2-Class	−62,502	125,561	40,669	125,130	40,888	6826	40,952	0.94	46%
3-Class	−56,534	113,907	11,654	113,257	11,873	6794	11,937	0.94	25%
4-Class	−54,963	111,048	2859	110,180	3078	6762	3142	0.92	16%
5-Class	−53,875	109,155	1893	108,068	2111	6730	2175	0.92	13%
6-Class	−53,000	107,688	1467	106,383	1685	6698	1749	0.92	6.8%
7-Class	−52,566	107,102	586	105,578	805	6666	869	0.91	6.0%
8-Class	−52,218	106,688	414	104,945	633	6634	697	0.88	5.6%
9-Class	−51,933	106,403	286	104,440	505	6602	569	0.88	4.5%
10-Class	−51,763	106,345	58	104,164	277	6570	341	0.87	1.5%
11-Class	−51,620	106,341	3	103,941	222	6538	286	0.87	1.5%
12-Class	−51,499	106,380	−39	103,763	178	6498	242	0.86	1.4%

Based on model selection statistics, a comparison of LCA models with different latent classes was made. LCA, Latent class analysis; LL, Log-likelihood; BIC, Bayesian information criterion (lower values imply better model fit); AIC, Akaike information criterion (lower values imply better model fit); df, degree of freedom; VLMR, Vuong–Lo–Mendell–Rubin likelihood ratio test; Entropy, higher values imply better classification quality.

**Table 3:** Class sizes and item-response probabilities at discharge.

	Class 1	Class 2	Class 3	Class 4	Class 5	Class 6	Class 7	Class 8	Class 9	Overall
**Class size**	29%	15%	11%	10%	10%	9%	6%	6%	4%	
**Discharge destination**										
Discharged home	**97%**	4%	46%	30%	35%	7%	**64%**	5%	9%	46%
Discharged to another hospital	1%	**77%**	**52%**	**61%**	**60%**	**82%**	33%	**91%**	**81%**	48%
Discharged to a care facility	1%	7%	1%	6%	4%	7%	1%	3%	7%	4%
In-hospital death	0%	7%	0%	0%	0%	1%	0%	0%	0%	1%
Others	1%	5%	1%	3%	1%	3%	2%	1%	3%	2%
**Length of stay**										
Less than 2 weeks (1 to 14 days)	**55%**	8%	6%	16%	19%	9%	21%	5%	6%	24%
2–4 weeks (15–28 days)	35%	19%	**50%**	40%	40%	28%	42%	33%	34%	35%
4–6 weeks (29–42 days)	7%	28%	25%	24%	27%	29%	21%	36%	32%	21%
More than 6 weeks (more than 42 days)	3%	44%	19%	21%	14%	35%	16%	27%	28%	20%
**NIHSS score**										
Right Motor Arm										
0	**97%**	20%	**85%**	**74%**	**79%**	**54%**	**91%**	**77%**	40%	**72%**
1	3%	9%	11%	17%	13%	17%	5%	5%	15%	9%
2	0%	16%	2%	4%	4%	13%	1%	5%	14%	6%
3	0%	17%	2%	4%	3%	9%	1%	9%	16%	6%
4	0%	37%	1%	1%	1%	7%	1%	4%	15%	8%
Left Motor Arm										
0	**96%**	24%	**81%**	**77%**	**72%**	37%	**96%**	35%	**84%**	**70%**
1	3%	11%	12%	15%	14%	12%	2%	17%	11%	9%
2	0%	15%	3%	4%	6%	14%	1%	16%	4%	6%
3	0%	16%	2%	3%	6%	16%	0%	19%	0%	6%
4	0%	35%	1%	1%	2%	20%	0%	14%	0%	8%
**FIM item**										
Eating										
Independence	**100%**	1%	**96%**	**51%**	**83%**	9%	**93%**	44%	22%	**62%**
Modified dependence	0%	5%	3%	42%	16%	45%	7%	39%	45%	16%
Complete dependence	0%	**95%**	1%	7%	1%	47%	0%	17%	34%	22%
Transfers (bed/chair/wheelchair)										
Independence	**99%**	0%	**98%**	3%	12%	0%	**93%**	0%	1%	47%
Modified dependence	1%	3%	2%	**97%**	**88%**	34%	7%	**73%**	**96%**	31%
Complete dependence	0%	**97%**	0%	0%	0%	**66%**	0%	27%	3%	22%
Toileting										
Independence	**100%**	0%	**95%**	3%	23%	0%	**87%**	0%	0%	47%
Modified dependence	0%	0%	5%	**86%**	**73%**	4%	13%	31%	46%	21%
Complete dependence	0%	**100%**	0%	11%	3%	**96%**	0%	**69%**	**54%**	32%
Locomotion (walking/wheelchair)										
Independence	**97%**	0%	**78%**	1%	4%	0%	**67%**	1%	0%	41%
Modified dependence	3%	1%	22%	**72%**	**81%**	3%	32%	13%	44%	23%
Complete dependence	0%	**99%**	1%	27%	15%	**97%**	1%	**86%**	**56%**	36%
Transfers (shower/bathtub)										
Independence	**75%**	0%	39%	0%	0%	0%	32%	0%	0%	28%
Modified dependence	18%	0%	**56%**	43%	**68%**	1%	**57%**	10%	24%	27%
Complete dependence	7%	**100%**	5%	**57%**	32%	**99%**	11%	**90%**	**76%**	45%
Communication (comprehension)										
Independence	**99%**	0%	**100%**	5%	**96%**	5%	9%	**91%**	2%	**56%**
Modified dependence	1%	8%	0%	**92%**	4%	**91%**	**87%**	9%	43%	27%
Complete dependence	0%	**92%**	0%	2%	0%	4%	4%	0%	**55%**	17%
Communication (expression)										
Independence	**99%**	0%	**93%**	8%	**93%**	3%	13%	**89%**	0%	**55%**
Modified dependence	1%	1%	6%	**90%**	6%	**84%**	**77%**	11%	20%	25%
Complete dependence	0%	**99%**	0%	2%	0%	13%	10%	0%	**80%**	21%
Social interaction										
Independence	**97%**	1%	**93%**	19%	**82%**	11%	38%	**72%**	11%	**56%**
Modified dependence	3%	4%	7%	**65%**	17%	**56%**	**54%**	25%	35%	22%
Complete dependence	0%	**95%**	0%	16%	1%	33%	8%	3%	**54%**	22%

FIM, Functional Independence Measure (range, 18–126, higher = better); NIHSS, National Institutes of Health Stroke Scale (range, 0–42, higher = worse). Bold numbers indicate class membership probabilities >50%.

## Data Availability

The data used in this study were obtained from the Japan Association of Rehabilitation Database under a data use agreement that prohibits sharing with third parties. Therefore, the data are not publicly available.

## Supplementary Materials

The following supporting information can be downloaded at: https://www.mdpi.com/article/10.3390/jcm14155466/s1, Table S1: Model fit of the prediction model; Table S2: Odds ratios (ORs) and *p*-values from the multivariate prediction of class membership (*n* = 6881); Video S1: Model application simulation.

## Abbreviations

The following abbreviations are used in this manuscript:

ADL	activities of daily living
LCA	latent class analysis
FIM	functional independent measure
NIHSS	National Institutes of Health Stroke Scale
STROBE	Strengthening the Reporting of Observational Studies in Epidemiology
JARD	Japan Association of Rehabilitation Database
AIC	Akaike information criterion
BIC	Bayesian information criterion
